# HSP90 inhibition blocks ERBB3 and RET phosphorylation in myxoid/round cell liposarcoma and causes massive cell death *in vitro* and *in vivo*

**DOI:** 10.18632/oncotarget.6336

**Published:** 2015-11-16

**Authors:** Setareh Safavi, Sofia Järnum, Christoffer Vannas, Sameer Udhane, Emma Jonasson, Tajana Tesan Tomic, Pernilla Grundevik, Henrik Fagman, Magnus Hansson, Zeynep Kalender, Alexandra Jauhiainen, Soheila Dolatabadi, Eva Wessel Stratford, Ola Myklebost, Mikael Eriksson, Göran Stenman, Regine Schneider Stock, Anders Ståhlberg, Pierre Åman

**Affiliations:** ^1^ Sahlgrenska Cancer Center, Institute of Biomedicine, Department of Pathology, Sahlgrenska Academy, University of Gothenburg, Gothenburg, Sweden; ^2^ Mathematical Statistics, Mathematical Sciences, Chalmers University of Technology and the University of Gothenburg, Göteborg, Sweden; ^3^ Department of Tumour Biology, The Norwegian Radium Hospital, Oslo University Hospital, Nydalen, Oslo, Norway; ^4^ Department of Oncology, Lund University Hospital, Lund, Sweden; ^5^ Experimental Tumor Pathology, Institute of Pathology, University of Erlangen-Nürnberg, Ulmenweg Erlangen, Germany

**Keywords:** sarcoma, receptor tyrosine kinases, HSP90 inhibitors, xenografts

## Abstract

Myxoid sarcoma (MLS) is one of the most common types of malignant soft tissue tumors. MLS is characterized by the *FUS-DDIT3* or *EWSR1-DDIT3* fusion oncogenes that encode abnormal transcription factors. The receptor tyrosine kinase (RTK) encoding *RET* was previously identified as a putative downstream target gene to *FUS-DDIT3* and here we show that cultured MLS cells expressed phosphorylated RET together with its ligand Persephin. Treatment with RET specific kinase inhibitor Vandetanib failed to reduce RET phosphorylation and inhibit cell growth, suggesting that other RTKs may phosphorylate RET. A screening pointed out EGFR and ERBB3 as the strongest expressed phosphorylated RTKs in MLS cells. We show that ERBB3 formed nuclear and cytoplasmic complexes with RET and both RTKs were previously reported to form complexes with EGFR. The formation of RTK hetero complexes could explain the observed Vandetanib resistence in MLS. EGFR and ERBB3 are clients of HSP90 that help complex formation and RTK activation. Treatment of cultured MLS cells with HSP90 inhibitor 17-DMAG, caused loss of RET and ERBB3 phosphorylation and lead to rapid cell death. Treatment of MLS xenograft carrying Nude mice resulted in massive necrosis, rupture of capillaries and hemorrhages in tumor tissues. We conclude that complex formation between RET and other RTKs may cause RTK inhibitor resistance. HSP90 inhibitors can overcome this resistance and are thus promising drugs for treatment of MLS/RCLS.

## INTRODUCTION

Myxoid liposarcoma (MLS) accounts for more than a third of all liposarcoma cases [1]. This entity is characterized by t(12;16) or more rarely t(12;22) chromosome translocations that result in fusions of the transcription factor gene *DDIT3* (also known as *CHOP* or *GADD153*) to *FUS* (also designated *TLS*) or to *EWSR1* [2–4]. The chimerical *FUS-DDIT3* and *EWSR1-DDIT3* encoded proteins are believed to function as abnormal transcription factors and *FUS-DDIT3* was reported to cause MLS like tumors in experimental mouse models [3, 5]. Most MLS tumors carry normal and functional *TP53* genes and are genetically stable [6, 7].

A subtraction screen for target genes downstream of *FUS-DDIT3* identified the proto oncogene *RET* among genes that are expressed in *FUS-DDIT3* carrying MLS, but not in normal adipose tissue or in benign lipoma [8]. *RET* encodes a receptor tyrosine kinase (RTK) [9–11] that may bind four alternative RET ligands. Ligand binding promotes RET complex formation, auto-phosphorylation [12] and activation of downstream signaling pathways [13–16]. RET can also form active heterodimers with the epithelial growth factor receptor (EGFR) [17] and recently the receptor tyrosine kinase MET was also reported to form heterodimers with RET in MLS tissues [18]. Such hetero-complex formation and activation of RET may operate in MLS cells as EGFR is strongly expressed in the tumor cells [19]. The observed co-expression of RET and EGFR in MLS prompted further investigation of RTKs as possible drug targets and their role in tumor development.

In the present study, we analyzed the expression of RET and its ligands in 8 MLS cases and 4 MLS derived cell lines and investigated its potential interaction with other RTK species. RTK inhibitors were tested for growth/survival effects in MLS cell lines. The HSP90 chaperone proteins enlist RET and several other RTKs as client proteins [20, 21]. HSP90 inhibitors were thus tested for effects in MLS cell lines and in an MLS xenograft model.

## RESULTS

### RET mRNA and protein is expressed in MLS cell lines and tumor tissues

A meta-analysis of public expression array datasets from 434 human tumor samples, pointed out expression of *RET* as typically for MLS compared to other soft tissue tumors (Supplementary Data Table 1).

**Figure 1:**
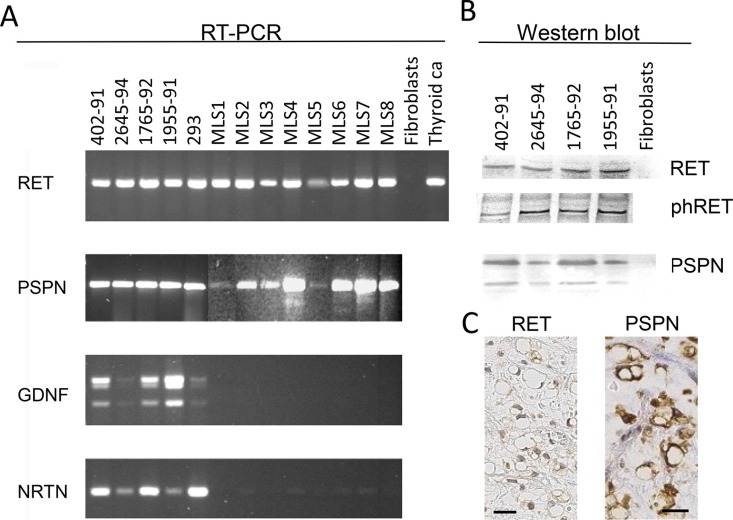
Expression of RET and ligands in MLS cell lines and tissues (**A**) RT-PCR analysis of the tyrosine kinase encoding parts of the RET transcript and of ligands PSPN, GDNF and NRTN. Lanes 402–91, 2645–94, 1765–92 and 1955–91 are MLS derived cell lines, 293 is a short term cultured thyroid cancer, MLS 1–8 are material from MLS tumor tissues. Thyroid Ca is thyroid carcinoma tumor tissue sample used as positive control. Fibroblasts were used as negative control as they were previously found to be negative for *RET* expression [11]. (**B**) Western blot analysis of MLS cell lines 402–91, 2654–94, 1765–91 and 1955–91 and negative control fibroblasts. Antibodies against RET, RET phospho-Y905 and RET ligand PSPN C-terminal. (**C**) Immunohistochemistry of sections from one MLS case, representative of 8 tested. Sections stained with antibodies specific for the RET and PSPN proteins. Exclusion of the primary antibody abolished all staining of the sections. Size bars are 20 μm.

RT-PCR analysis with three primer pairs that amplified sequences encoding extracellular, transmembrane and tyrosine kinase encoding parts showed that full length *RET* transcripts were expressed in all MLS tumors investigated (Figure 1A). Western blot analysis of RET in protein extracts from three MLS cell lines showed reactive bands at 170 kDa, the reported size for the longest RET isoform (Figure 1B), and immunohistochemical (IHC) analysis of tumor tissues detected RET protein in both nuclei and cytoplasm of most tumor cells (Figure 1C).

### The RET protein is phosphorylated in MLS cells

Ligands or activating mutations cause autophosphorylation of RET Y905 [22]. At least a fraction of the RET molecules reacted with a phospho-Y905 specific antibody in MLS (Figure 1B). Sequencing of four MLS cell lines showed that the Y905 phosphorylation was not caused by activating mutations in *RET* [6]. Other full exome sequencing efforts on large cohorts of MLS also report normal *RET* gene sequences [7].

### MLS tumors express the RET ligand Persephin but not co-receptors GFRα1–4

Activation of RET could be caused by RET ligands in MLS. Our analysis of MLS cell lines showed that they contained mRNAs for glia derived neurotrophic factor (GDNF), neurturin (NRTN) and persephin (PSPN) but RNA extracted from tumor tissues contained only the *PSPN* transcript (Figure 1A). Sequence analysis of *PSPN* cDNA from MLS cell lines showed a normal *PSPN* sequence (data not shown) and Western blot analysis of protein extracts from MLS cells showed two bands at the sizes reported for the unprocessed PSPN precursor (17 kDa) and the mature PSPN (12 kDa) (Figure 1B). Both bands were detected with antibodies against N-terminal and C-terminal parts of PSPN. IHC analysis with PSPN specific antibodies in sections from eight MLS tumors showed a cytoplasmic staining of the tumor cells in all cases (Figure 1C). RT-PCR analysis of RET co-receptors GFRα1–4 in four MLS tumors and three MLS-cell lines detected no transcripts (data not shown).

### RET RTK inhibitor Vandetanib fails to block RET Y905 phosphorylation and cell growth/survival of MLS derived cells lines

The RTK inhibitor Vandetanib is reported to inhibit RET kinase activity with 50% inhibition at 100 nM [23]. However, treatment of MLS cells with up to 2 μM Vandetanib failed to reduce RET Y905 phosphorylation and cell proliferation/survival (Supplementary Figure 1A and 1B). The observed phosphorylation of RET Y905 may therefore depend on other, Vandetanib-resistant kinases.

### Phosphorylated EGFR and ERBB3 are expressed in MLS cells and tissues

**Figure 2:**
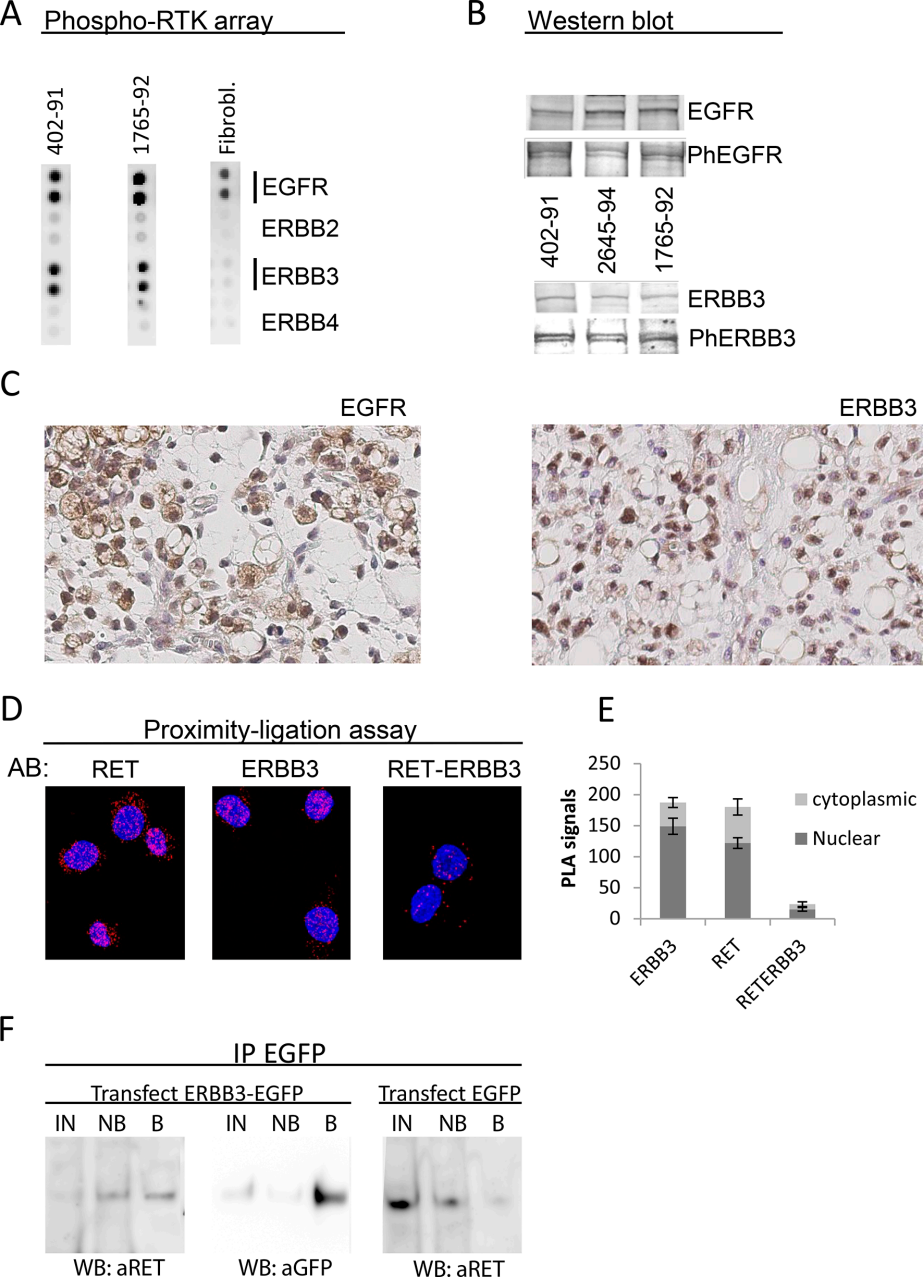
Expression of EGFR and ERBB3 in MLS cell lines and tissues (**A**) RTK phospho-array analysis of MLS 402–91 and 1765–92 cell lines. Cultured normal fibroblasts as control sample. (**B**) Western blot analysis of EGFR, EGFR phospho-Y1068, ERBB3 and ERBB3 phospho-Y1289 in MLS cell lines 402–91, 2645–94, 1765–92. (**C**) Immunohistochemistry of sections from one MLS case representative of 8. Sections stained with antibodies specific for the EGFR and ERBB3 proteins. Exclusion of the primary antibody abolished all staining of the sections. Size bars are 20 μm. (**D**) PLA analysis of RET-ERBB3 interaction. AB: indicates specificity of antibodies used. Red dots indicate signals from antibodies that are located close enough for the ligation assay to score. The RET and ERBB3 panels shows signals from antibodies targeting RET and ERBB3, respectively, and the RET-ERBB3 panel shows signals from interactions between one RET and one ERBB3 antibody as described in materials and methods. (**E**) Quantification of PLA signals from RET-RET, ERBB3-ERBB3 and RET-ERBB3 antibody combinations in cytoplasm and nuclei. Mean numbers of signals and standard error of means shown from five randomly selected cells of each experiment. (**F**) Co-immunoprecipitation of RET protein with EGFP antibodies in ERBB3-EGFP and EGFP transfected MLS cell line 1765–92. Western blot detection of precipitated proteins is shown. IN: Input proteins for IP, NB: non-bound proteins, B: precipitated proteins.

A dot blot screen for phosphorylated RTKs pointed out EGFR and ERBB3 as the most strongly expressed, phosphorylated RTKs in MLS cell lines (Figure 2A). Western blot analysis confirmed that EGFR (130 kDa) and ERBB3 (190 kDa) proteins were expressed in three MLS cell lines and that at least a fraction of these proteins were phosphorylated at their activation specific positions Y1068 and Y1289, respectively (Figure 2B). IHC studies of MLS tissues showed surface/cytoplasmic expression of EGFR whereas ERBB3 showed nuclear/cytoplasmic localization in MLS tissues and cell lines (Figure 2C).

### RET and ERBB3 proteins form complex in MLS cells

RET and EGFR as well as EGFR and ERBB3 are known to form hetero-complexes [17, 24]. Our IHC observation that ERBB3 and RET partially co-localizes (Figures 1C and 2C), raised our suspicion that RET and ERBB3 also could form complexes. Proximity ligation assays (PLA) showed that ERBB3 and RET localized close enough for PLA detection (Figure 2D), indicating that these molecules indeed form hetero-complex in MLS cells. The hetero-complex formation was confirmed by co-immunoprecipitation one word of RET with EGFP-antibodies in ERBB3-EGFP transfected MLS cells (Figure 2F).

Our IHC and PLA results showed a mixed cytoplasmic and nuclear expression of RET and ERBB3 (Figures 1C, 2C and Supplementary Film Clips).

### The EGFR tyrosine kinase inhibitor Gefitinib fails to inhibit cell growth/survival of MLS derived cells lines

EGFR specific Gefitinib failed to inhibit cell growth and survival of MLS cell lines at concentrations with maintained RTK specificity [25] (Supplementary Figure 1C). Combinations of Gefitinib and Vandetanib also failed to block growth/survival at concentrations within their range of specificity (Supplementary Figure 1D).

### The HSP90 inhibitor 17-DMAG causes loss of phosphorylated RET, EGFR and ERBB3 proteins in MLS cell lines

**Figure 3:**
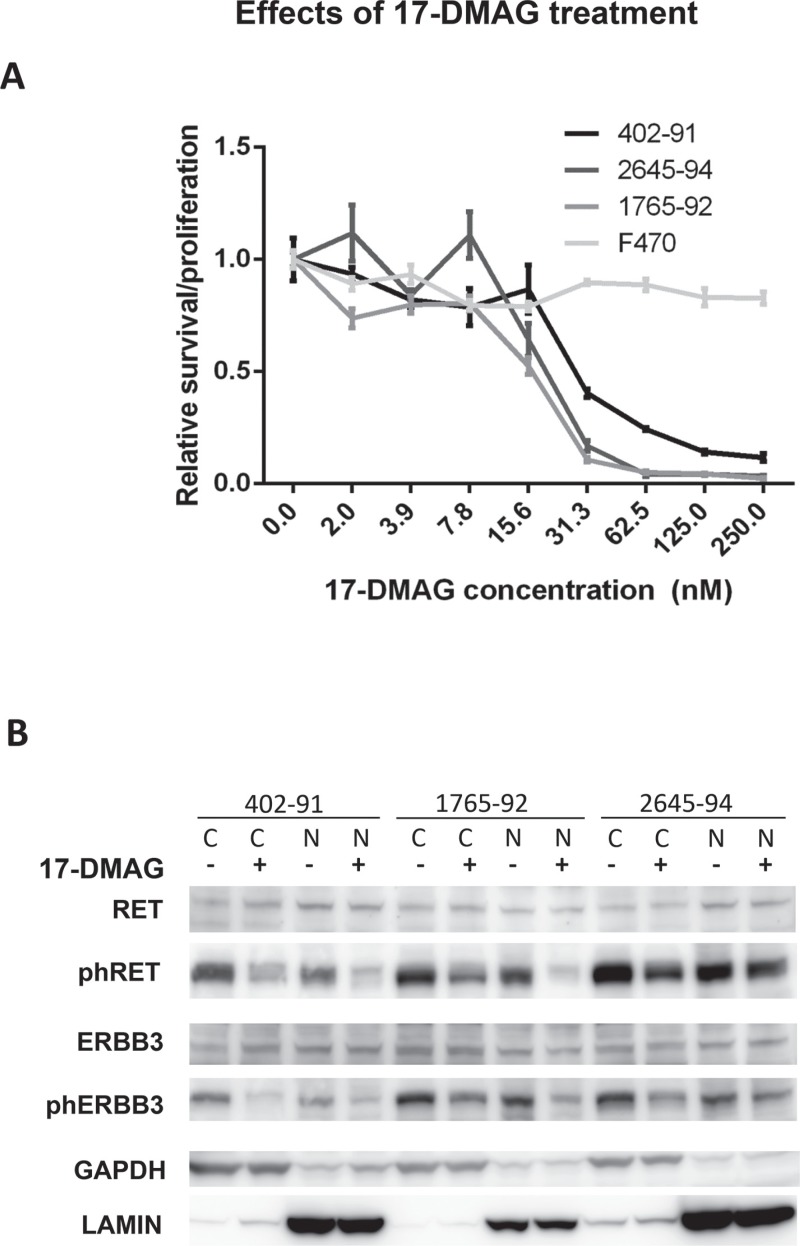
*In vitro* effects of 17-DMAG treatment (**A**) Cell survival after 17-DMAG treatment. Cell density measured after 72 hours treatment of MLS cell lines (402–91, 1765–92 and 2645–94) and normal fibroblasts (F470) with various 17-DMAG concentrations. Values normalized against untreated control cells. Standard error of mean, indicated as error bars, was calculated from four replicates in different wells of the plate. (**B**) Western blot analysis of total and phosphorylated RTKs extracted from cytoplasmic and nuclear fractions of MLS cell lines after 24 hours of treatment with 30 nM 17-DMAG as indicated. Nuclear and cytoplasmic fractions indicated by N and C respectively. Loading and fraction controls: GAPDH (cytoplasmic) and Lamin A (nuclear).

The failure to inhibit RET Y905 phosphorylation with specific tyrosine kinase inhibitors could possibly be explained by formation of multi-member protein complexes between RET, EGFR, ERBB3 and perhaps yet other tyrosine kinases. This hypothesis led us to investigate other means of modifying the activity of the RTKs. RET, EGFR and ERBB3 are listed among clients of HSP90 chaperone proteins [20, 21]. Cell proliferation and survival assays showed an LD50 between 10 and 30 nM for the HSP90 inhibitor 17-DMAG in the tested MLS cell lines (Figure 3A). The RET Y905, and ERBB3 Y1289 phosphorylation levels were reduced 24 hours after addition of 17-DMAG while the total RET and ERBB3 protein levels were unaffected (Figure 3B).

### The HSP90 inhibitor 17-DMAG causes massive necrosis in xenografted MLS tumors

**Figure 4:**
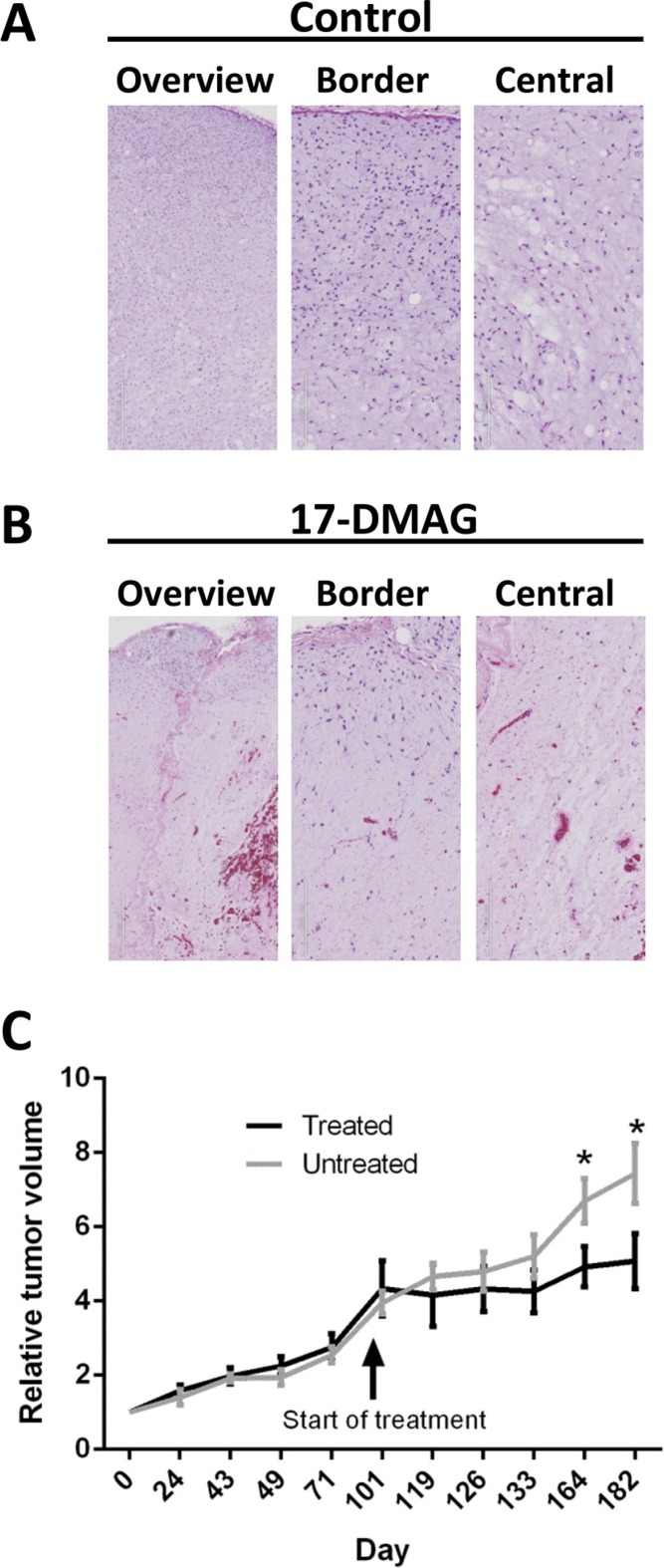
Histology of MLS xenografts from NUDE mice (**A**) Control tissue showing typical MLS features with prominent myxoid matrix, lipoblasts and capillary vessels. Overview panel at 4 times, and close up border and central zone panels at 12 times magnification. (**B**) Tumor tissue one week after 3 days of 17-DMAG treatment, 25 mg/Kg. Note extensive cell loss, rupture of capillary vessels and bleeding. See also microphotographs at higher resolution in Supplementary Figure 2. (**C**) Follow up on tumor volume in control and 17-DMAG treated animals. Time point for first injection at 100 days after first tumor measurement is indicated. Tumor growth was followed for 83 days after first injection. The mice were given three injections with 48 hours intervals, repeated for two weeks with a 72 hours break after injection 3. Data from 8 treated and 8 control tumors is shown as relative mean volumes using data from first measurements as base value. Standard error and significant differences according to *t*-test are shown as bars and stars.

An MLS tumor was serially transplanted in BALB/C NUDE mice and grew with the typical histological features of MLS (Figure 4A and Supplementary Figure 2A). Xenografts from two mice, each carrying two tumors, were treated for three days, one injection/day with 17-DMAG. Three of four tumors showed hemorrhages with scattered extravasated erythrocytes and extensive coagulation type necrosis of tumor cells as well as of small vessels. The fourth and smallest tumor showed smaller affected areas and no hemorrhages. Four tumors analyzed after two weeks of treatment showed similar morphological changes. The extent of necrosis remained with a peripheral rim of viable tumor cells and without evidence of adipogenic maturation or stromal hyalinization (Figure 4B and Supplementary Figure 2B).

In a separate experiment, 4 animals, each carrying two tumors were given three injections of 17-DMAG with 48 hours intervals. This was repeated for two weeks with a 72 hours interval after the third injection. This treatment caused no dramatic changes in tumor size during, or immediately after the treatment period, but most tumors decreased slowly in size or halted their growth during an 83 day follow up period (Figure 4C).

## DISCUSSION

Our previous search for possible downstream target genes of *FUS-DDIT3* identified *RET* as a candidate [8] and meta-analysis of microarray data in this study confirmed expression of *RET* as a characteristic of MLS (Supplementary Data Table 1). Expression of RET in MLS has independently been reported by others and higher expression level has been associated with poor survival [18, 26, 27]. Here, we show that PSPN, one GDNF-family member ligand of RET, is expressed in MLS and that the RET protein is phosphorylated at tyrosine 905 (Y905) as a sign of receptor activation [28–33]. PSPN binds to RET in complex with glycosyl-phosphatidylinositiol (GPI)-anchored co-receptors GFRα1–4 [13–15, 34, 35]. We did not detect any *GFR*α1–4 transcripts in the tumors or cell lines. However, GFRα1–4 independent ligand binding and RET signaling has been described [36], indicating a possible role for autocrine RET activation by PSPN in MLS. Expression of Artemin, another RET ligand and the RET coreceptor GFRα3 has been reported in MLS by Negri *et al.* [18], but we have, for unknown reasons, not detected transcripts/proteins from these genes in our cohort of MLS. Taken together, the expression of RET in MLS and the reported function of RET in cell survival/growth mechanisms, suggests that this RTK may be important in MLS pathogenesis. These results also provided a rationale for trying to block RET Y905 phosphorylation with the RET specific TK inhibitor Vandetanib. This drug failed, however, to block RET Y905 phosphorylation or cell growth indicating that RET phosphorylation was maintained by a Vandetanib resistant kinase, possibly another RTK species that could form complex with RET.

Our screen for phosphorylated RTKs pointed out EGFR and ERBB3 as the most abundantly expressed in MLS. Using PLA and co-IP techniques, we showed, for the first time, that RET and ERBB3 form hetero-complexes. ERBB3 forms hetero-complexes with several other members of the EGFR RTK family but cannot form homodimers [24]. Adding to the complicated scenario of interacting RTKs, a previous report also suggest that RET may interact with and be phosphorylated by the MET RTK in MLS [18]. MLS cells thus contain at least four distinct RTK species (RET, EGFR, MET and ERBB3) that can form various complexes around RET. This could result in deregulated RTK activation and increased growth/survival of the tumor cells.

The finding that RET may form dimers with EGFR [17], provided a rationale for trying to block RET Y905 phosphorylation and growth/survival with the EGFR specific RTK inhibitor Gefitinib or combinations of Gefitinib and Vandetanib. No decrease in cell growth was observed at concentrations within the range of RTK specificity, indicating that the survival/growth supporting activity was maintained by a Gefitinib and Vandetanib resistant mechanism. The cytoplasmic tyrosine kinase SRC has been reported to phosphorylate RET at Y905 and promote RET protein activity [37]. This SRC activity could possibly explain the Vandetanib resistance of RET in MLS is compatible with the reported growth inhibiting and cytotoxic effects of a specific SRC inhibitor in MLS cells [38].

Our IHC analysis showed that RET and ERBB3 was expressed as cytoplasmic/nuclear proteins and this was confirmed by PLA analysis (Figure 2D, 2E and Supplementary Film Clips 1–3) and western blot analysis of cytoplasmic and nuclear cell fractions (Figure 3B). Nuclear, smaller variants of ERBB3 have been reported to bind the promoter and activate transcription of the cyclin D1 gene (*CCND1*) [39]. We could not detect shorter ERBB3 variants in MLS but found the full-length protein in nuclear MLS extracts (Figure 3A). A role for nuclear ERBB3 in *CCND1* regulation is compatible with the reported strong expression of *CCND1* in MLS [19]. Nuclear expression and specific nuclear down-stream effects have been reported for many different RTK species including EGFR, ERBB3 and RET [40–43]. Nuclear RET expression was reported to cause phosphorylation of the apoptosis promoting transcription factor ATF4 and thus inhibit apoptosis and promote survival [43]. The nuclear expression of ERBB3 together with RET in MLS may thus be central for MLS pathogenesis.

We have previously reported a cytoplasmic/nuclear expression of the FLT1 receptor tyrosine kinase and its ligand PLGF in MLS [44]. Taken together, at least three RTK species (RET, ERBB3, FLT1)with two of their ligands (PSPN, PGF) show a nuclear localization in these cells. This unusual expression pattern stands out as typical for MLS compared to normal mesenchymal cells. Cross activation between these and perhaps additional tyrosine kinases may thus be an essential function for self-sustaining growth and survival of MLS cells and could also cause the observed resistance to RTK inhibitors [45].

RTK activation and complex formation has been reported to depend on HSP90 activity [45, 46]. Our data shows that inhibition of HSP90 functions lead to loss of RET and ERBB3 phosphorylation. It also caused growth cessation and death of MLS cells *in vitro* and extensive necrosis of xenografted MLS tumors. The cytostatic *in vitro* effects of HSP90 inhibition in MLS cell lines has recently been reported for an unrelated HSP90 inhibitor [47], confirming the MLS sensitivity to this class of drugs.

Several mechanisms for HSP90 inhibitor induced cell death have been described including inhibition/degradation of RTKs [45, 46, 48–51]. HSP90 has a large number of client proteins and inhibition can be expected to affect many cellular functions leading to cell death. However, normal cells, including the human fibroblasts used as *in vitro* controls here, were highly resistant to HSP90 inhibitors. An outstanding difference between normal fibroblasts and MLS cells, is the co-expression and nuclear expression of RET and ERBB3. This difference may play an important role in the vulnerability of MLS cells exposed by HSP90 inhibition. The fact that 17-DMAG caused decreased ERBB3 and RET phosphorylation with maintained total levels of these proteins prior to cell death supports this conclusion.

Our *in vitro* results lead us to try HSP90 inhibitor treatment in an MLS xenograft model. This tumor has been serially transplanted from the patient and was not passaged in culture. It closely recapitulates the typical histology and slow growth pattern of MLS tumors. HSP90 inhibitor treatment caused massive cell death and bleeding/rupture of tumor capillaries. Macroscopically, the tumor tissues showed a slow decline or in some cases stable size. Histological examination revealed narrow marginal regions of surviving tumor cells several months after finished treatment. These surviving tumor cells may be senescent since MLS tumors contain major populations of resting and senescent cells [52]. Senescent cells are still capable of maintaining the extensive extracellular matrix [53], and could therefore prevent fast shrinkage of 17-DMAG treated tumor tissues. The small and slow size reduction of MLS tumors stands in contrast to the dramatic histological findings and has to be taken into consideration when clinical trials with MLS patients are to be evaluated.

We conclude that MLS cells express a mix of interacting RTKs and their ligands that could cross interact and cause their resistance to RTK inhibitors. Inhibition of HSP90 activity could overcome this resistance as shown by reduced RTK phosphorylation/proliferation and massive death of the tumor cells *in vitro* and *in vivo*. HSP90 inhibitors are therefore attractive drug candidates for treatment of MLS.

## MATERIALS AND METHODS

### Tissue samples

The tumor material is summarized in Table 1. Formaldehyde fixed tissues were embedded in paraffin and fresh tissues were frozen and stored at −140°C.

**Table 1:** Cell lines and tissue samples

SAMPLE NUMBER	TUMOR TYPE	SAMPLE	FUS-DDIT3**
402–91	MLS	Cell line	+
1955–91	MLS	Cell line	+
2645–94	MLS	Cell line	+
1765–92	MLS	Cell line	+
Case1	MLS	Tissue	+
Case2	MLS	Tissue	+
Case3	MLS	Tissue	+
Case4	MLS	Tissue	+
Case5	MLS	Tissue	+
Case6	MLS	Tissue	+
Case7	MLS	Tissue	+
Case8	MLS	Tissue	+
Xenograft	MLS	Tissue	+

** Results from RT-PCR experiment

### Cell culture and cell lines

SV40 large T transformed myxoid liposarcoma cell lines 1955–91, 1765–92, 402–91 and 2645–94 [54–56], normal human fibroblasts F470, passages 4–8, MCF-7 (human breast cancer line), HT1080(human fibrosarcoma cell line [57]), were cultured in RPMI1640 with 8% fetal calf serum. All media and supplements were obtained from Life Technologies. Transfections were made with FUGENE 6 reagent (Promega) according to the manufacturers protocoll. ERBB3 cDNA clone (Addgene 23874) was inserted into a pEGFP-N1 vector (Clontech) and one control sequenced clone was used for transfection.

### Proliferation and cytotoxicity assay

Cells were seeded to 96-well microtiter plates. Drugs were added after 24 hours at different concentrations. Each test was set up in three or more replicates. The medium was removed and plates were washed 72 hours after treatment and stored in −80°C until analysis. The cells were lysed and DNA was stained using a Cell Proliferation Assay Kit (Molecular probes, C–7026) according to the manufacturers manual, and analyzed in a SpectraMaxGeminiXS (Göteborgs Termometerfabrik Gothenburg Sweden) or in a VICTOR^3^ multilabel plate reader (PerkinElmer).

### Nucleic acid extractions

DNA was extracted from frozen tumor tissues as described [2]. RNA was extracted from tumor tissues using a Fastprep kit (Bio101) according to the manufacturer's recommendations. RNA was extracted from cultured cells as described [58].

### RT-PCR analysis

RT-PCR analysis was performed with the following *RET* primers: RET225 5′-GGCTGCGTCTGCTGTGC TG and RET466 5′-AAGGTGAGGAGGCCGGTGTC, covering sequences encoding extracellular parts, RET 2185 5′-GCCCACAGCCACCCAT and RET 2469 5′-GGA GGCGTTCTCTTTCAGCATC, covering transmembrane encoding parts, RET2738 5′-TGGGCGACCTCATCTC ATTTG and RET3271 5′-AGGCCGTCGTCATAAATC AGGGAG, covering the tyrosine kinase encoding sequences of *RET* mRNA. For the analysis of ligand transcripts, the *GDNF* specific primers GDNFfwd 5′-ATGTCGTGGCTGTCTGCCTGG and GDNFrev 5′-CATCGCAAGAGCCGCTGCAG, the *PSPN* specific primers PSPN90fwd 5′-CGTGGCCGATGGAGAGTT CTC and PSPNrev 5′-AAGGCCACGTCGGTGTAGCG, the *NRTN* specific primers NTNfwd 5′-AGAGGGCC TGCTTCTCAGCC and NTNrev 5′-TAGCGGAACAGC ACCGTCTCG, and the *ARTN* specific primers ARTNfwd 5′-TGCTGAGCAGCGTCGCAGAGG and ARTNrev 5′-AGGAGCCGCTGCAGAAGCGG, were used. To avoid amplification of contaminating genomic DNA, all primer pairs were designed to amplify cDNA fragments that spanned over one or several exon borders. Unigene representative sequences were used as template for primer design. PCR was run for 35 cycles using a GCRICH PCR system (Roche Diagnostics) and AmpliTaq GOLD Polymerase (Perkin-Elmer) according to the manufacturer's recommendations. PCR detection of *FUSDDIT3* fusion transcripts was performed as previously described [4, 56].

### Mutation analysis

Exons 10 and 11 of the RET gene were amplified from genomic tumor DNA by PCR using the following primers: CRT19S 5′-GCAGCATTGTTGGGGGACA (exon 10, forward), CRT2A 5′-GACAGCAGCACCGAGACGAT (exon 10, reverse), MENF 5′-CATGAGGCCGA GCATACTCAGCC (exon 11, forward), MENR 5′-CA GACAGCAGCGCCGAGACGATG (exon 11, reverse). Amplification was accomplished by 35 cycles using the following parameters: denaturation 94°C for 45 seconds, annealing 56°C for 90 seconds, extension 72°C for 90 seconds. PCR products were purified on Microspin S300 HR columns (Pharmacia Biotech, Uppsala, Sweden). An aliquot (5–10 ml) of the purified PCR product served as template in a Big Dye Terminator Cycle Sequencing Ready Reaction procedure (PE-biosystems no 4303152) and the product was analyzed in an ABI PRISM 377 DNA sequencer (Perkin Elmer Biosystems, Foster City, CA). Forward primers were used for the sequencing procedure and each sample was analysed in at least three separate reactions. Sequences were analysed using the Sequence Navigator software.

### Immunohistochemistry (IHC)

Sections were cut from blocks with formaldehyde fixed paraffin embedded tumor tissues, deparaffinized and stained with primary antibodies specific for RET and PSPN, (Santa Cruz Biotechnology, product numbers SC-167 and SC-8684, respectively). The RET antibody was used at 1:100 dilutions whereas the PSPN antibody was applied at a 1:20 dilution. Exclusion or replacement of primary antibodies from the IHC protocol abolished all staining of the tissue sections.

### Proximity ligation assay

The proximity ligation assays was performed with a DuoLink II kit(Olink Bioscience) according to the manufacturers protocols and as described [58]. DuoLink rabbit and goat specific probes were used in combination with ERBB3 specific rabbit antibody (G-4) (1:300) and RET specific goat antibody (C-19) (1:200), both from Santa Cruz Biotechnology. Assays with unmatched first antibodies and DuoLink probes and with single DuoLink probes were used as negative controls and gave no background signals.

Microphotographs were captured on a Zeiss LSM 700 confocal microscope and deconvolution, image analysis, quantification of PLA spots and animation clips were made with the Volocity soft-ware package, (PerkinElmer Inc). The deconvolution was performed using iterative restoration with a calculated point spread function.

### Western blots analysis

Whole cell extracts were prepared as described [58] and separated on a 4–12% SDS-PAGE gel (Life Technologies as previously described [59]. The proteins were blotted on Immobilon™-P PVDF membranes (Millipore Corporation) or Invitrolon PVDF membranes (Life Technologies) and detected using the following antibodies: RET (C-19), goat polyclonal (Santa Cruz Biotechnology); phospho-RET Tyr-905, rabbit polyclonal (Cell Signaling Technology); Persephin (N18 and C17), goat antisera (Santa Cruz Biotechnology); EGFR (E30), mouse monoclonal (DAKO); phospho-EGFR Tyr-1068 (1H12), mouse monoclonal (Cell Signaling Technology); ERBB3 (G-4), mouse monoclonal (Santa Cruz Biotechnology); phospho-ERBB3 Tyr-1289 (21D3), rabbit monoclonal (Cell Signaling Technology); GFP (JL-8) mouse monoclonal (Clontech); GAPDH (mAbcam 9484), mouse monoclonal (Abcam); Lamin A (133A2), mouse monoclonal (Abcam). RP-conjugated secondary antibodies were used: Stabilized Goat Anti-Mouse IgG (H + L), Peroxidase Conjugated (Thermo Scientific Pierce); Stabilized Goat Anti-Rabbit IgG (H + L), Peroxidase Conjugated (Thermo Scientific Pierce); bovine anti-goat IgG-HRP (Santa Cruz Biotechnology).

All antibodies were evaluated by tests on positive and negative control western blot samples and normal human tissue sections with published and established expression patterns of EGFR, ERBB3 and RET in specific cell types as described [19]. Extracts from human cultured fibroblasts were used as control for RET, Persephin and ERBB3 proteins. Breast cancer cell line MCF-7 was used as positive control of ERBB3.

Preparation of cytoplasmic and nuclear extracts was made from trypsin detached cell suspensions that were washed twice in PBS and suspended in 100 μl of ice-cold lysis buffer containing 50 mM NaCl, 4 mM MgCl2, 10 mM TRIS PH 6. 8, 0, 5% TritonX. The cells were disrupted by repeated pipetting for 5 minutes on ice. Nuclei were pelleted by centrifugation at 14000 rpm for 15 minutes. Cytoplasmic (supernatant) and nuclear (pellet) fractions were collected and diluted 1: 4 with 4 × NuPAGE LDS sample buffer (Life technologies) for Western blot analysis. Immunoprecipitation of GFP tagged ERBB3 was made 24 hours after transfection. Cells were harvested and suspended in lysis buffer (25 mM Tis pH 7.5, 150 mM KCl, 1,5 mM MgCl, 1 mM DTT, and 0,5% NP 40. After 30 minutes on ice followed by a 10 minutes centrifugation at 16000 × G, the supernatant was diluted to 0,2% NP40. Immunoprecipitation was performed with GFP-TRAP beads (Chromotek) according to the manufacturers protocol.

### Animals and drug treatment

Five to six weeks old BALB/C NUDE mice (Janvier Labs) were transplanted with approximately 2 × 2 mm large tumor pieces. Mice with tumor sizes from 8 × 8 mm were used for drug treatment tests. Tumor sizes were measured in mm with a caliper from 14 days after transplantation and volume calculated according to Feldman *et al.* [60]. 17-DMAG (Selleckchem) was dissolved in 150 mM NaCl to a final concentration of 1 mg/ml and stored frozen. The drug was administered by peritoneal injections (25 mg/Kg body weight). Drug treatment was started 114 days after transplantation with three injections at 48 hours intervals repeated for two weeks with a 72 hours break after injection 3. Tumor sizes were followed up for 83 days after first injection. The mice were sacrificed by cervical dislocation and tumors were immediately collected and transferred to ice-cold 4% formaldehyde in PBS.

## SUPPLEMENTARY TABLES, FIGURES AND MOVIES








