# Supplementary material for: HSP90 inhibition blocks ERBB3 and RET phosphorylation in myxoid/round cell liposarcoma and causes massive cell death in vitro and in vivo
Source: Oncotarget. 2015 Nov 16;7(1):433–45. doi: 10.18632/oncotarget.6336 (PMC4808009; doi:10.18632/oncotarget.6336)
Supplement: Supplementary file 1 [file oncotarget-07-0433-s001.pdf]

HSP90 inhibition blocks ERBB3 and RET phosphorylation in myxoid/round cell liposarcoma and causes massive cell death *in vitro* and *in vivo*

Supplementary Materials

A gene expression meta-signature for myxoid liposarcoma.

The hypothesis addressed here is that if a meta-signature existed, the genes in the signature would reflect the essential transcriptional features of myxoid liposarcoma (MLA). There are 26 signatures analyzed for this case. At the significance threshold of Q-value less than 0.05, 87 genes were present in at least 22 of the 26 studies with minimum false discovery rate of 0.0714. All of the genes existing in this meta-signature are listed in Table S1**.**

We have used the *limma* package in R/Bioconductor platform to calculate moderated t-statistics and *p*-value*s* (1). Multiple testing issue was addressed by calculating *q*-values (2). To identify the meta-signature, we have modified and implemented the procedure described in (3) as follows:

1. A set S of differential expression analyses were selected and a significance threshold T was chosen to define differential expression signatures from the selected analyses ($T_{default}$ = 0.10). Genes with a q-value below the threshold T were selected.
2. Genes were sorted by the number of signatures they were present in.
3. The number of genes present in each possible number of signatures was tallied (N_1, N_2 , ..., N_{26}).
4. Random permutations were performed in which the actual q-values were assigned randomly to genes per signature, so that the genes in each signature changed randomly, but the number of genes in each signature remained the same. Randomization pattern was the same between signatures ensuring the dependence of genes across signatures during the randomization process. This simulation generated a tally of number of genes present in each possible number of random signatures (E_1, E_2, ..., E_{26}).
5. The significance of intersection among the true signatures was assessed by the minimum meta-false discovery rate (mFDRmin) as:

   $mFDR_{min} = \min([E_i + 1]/[N_i])$ for $i = 1$ to 26
6. If $mFDR_{min}$ was less than 0.10, a meta-signature was defined as those genes that were significantly differentially expressed ($q \leq T$) in at least j of S analyses, where j was equal to *i* when $mFDR_{min}$ was defined.
7. If no meta-signature was defined by using $T_{default}$, steps 2 through 6 were repeated where T was lowered by 50% at each iteration until either a meta-signature was defined or the number of genes in two or more signatures reached zero.

The expression rate in Table S1 was defined as:

Nummer om signature in which the is gene was significantly regulated / Number of signatures in which the gene was upregulated

For example, *AGT* displayed an expression rate of 26/26, which means that *AGT* was upregulated in all signatures. *GYG2* on the other hand showed an expression rate of 22/19, which means that the gene was differently expressed in 22 of 26 signatures. Nineteen of these 22 regulates were upregulations.

In the original method, the random signatures were generated without considering the dependency of genes across different comparisons. In our method, the same randomization pattern was applied to signatures that were generated from the same dataset, enabling a more realistic simulation. Another modification was to use moderated t-statistic instead of standard t-statistic when calculating *p*-values, thus enhancing the robustness of the test statistic.

Two public microarray datasets were analyzed to identify a meta-signature for MLS (Table S2). These datasets contained a range of sarcomas, including MLS, and we aimed to find a meta-signature of expressed genes for MLS by performing a two class differential expression analysis with MLS versus each of the different tumor types. In total, we have performed 26 comparisons, thus generated 26 signatures (Table S3).

Table S1: A gene expression meta-signature for myxoid liposarcoma

Gene symbol	Gene name	Expression rate
AGT	angiotensinogen (serpin peptidase inhibitor)	26/26
AKAP1	A kinase (PRKA) anchor protein 1	26/26
CLK4	CDC-like kinase 4	26/26
CTAG2	cancer/testis antigen 2	26/26
EBF2	early B-cell factor 2	26/26
EMX2	empty spiracles homeobox 2	26/26
FAM13A1	family with sequence similarity 13, member A	26/26
HOXA5	homeobox A5	26/26
HSD11B2	hydroxysteroid (11-beta) dehydrogenase 2	26/26
MYH15	myosin, heavy chain 15	26/26
OPRK1	opioid receptor, kappa 1	26/26
PTH2R	parathyroid hormone 2 receptor	26/26
PTX3	pentraxin-related gene, rapidly induced by IL-1 beta	26/26
RAB11FIP2	RAB11 family interacting protein 2 (class I)	26/26
SHANK2	SH3 and multiple ankyrin repeat domains 2	26/26
SIM1	single-minded homolog 1 (Drosophila)	26/26
CA4	carbonic anhydrase IV	25/25
HOXA4	homeobox A4	25/25
IDH1	isocitrate dehydrogenase 1 (NADP +), soluble	25/25
KLHDC8A	kelch domain containing 8A	25/25
PPARG	peroxisome proliferator-activated receptor gamma	25/25
RET	**ret proto-oncogene**	**25/25**
SAMM50	sorting and assembly machinery component 50	25/25
SCPEP1	serine carboxypeptidase 1	25/25
SH3PXD2A	SH3 and PX domains 2A	25/25
SLCO1C1	solute carrier organic anion transporter 1C1	25/25
TAC1	tachykinin, precursor 1	25/25
CD36	CD36 molecule (thrombospondin receptor)	24/24
CIB2	calcium and integrin binding family member 2	24/24
FAM65B	family with sequence similarity 65, member B	24/24
FZD4	frizzled homolog 4 (Drosophila)	24/24
GPD1	glycerol-3-phosphate dehydrogenase 1 (soluble)	24/21
KCNJ3	potassium channel, subfamily J, member 3	24/24
MAN2A2	mannosidase, alpha, class 2A, member 2	24/24
MAPK10	mitogen-activated protein kinase 10	24/24
MOSC1	MOCO sulphurase C-terminal domain containing 1	24/21
NMNAT2	nicotinamide nucleotide adenylyltransferase 2	24/24
NRG2	neuregulin 2	24/24

PCNX	pecanex homolog (Drosophila)	24/24
RERE	arginine-glutamic acid dipeptide (RE) repeats	24/24
SOX11	SRY (sex determining region Y)-box 11	24/24
ACACB	acetyl-Coenzyme A carboxylase beta	23/23
ACO1	aconitase 1, soluble	23/23
ANGPT1	angiopoietin 1	23/23
C1orf115	chromosome 1 open reading frame 115	23/23
CITED1	Cbp/p300-interacting transactivator 1	23/23
CTAG1B	cancer/testis antigen 1B	23/23
FGFR2	fibroblast growth factor receptor 2	23/23
FMO2	flavin containing monooxygenase 2 (non-functional)	23/23
GNAT3	guanine nucleotide binding protein, alpha transducing 3	23/23
HSDL2	hydroxysteroid dehydrogenase like 2	23/23
ITIH5	inter-alpha (globulin) inhibitor H5	23/23
LIPE	lipase, hormone-sensitive	23/20
PGRMC2	progesterone receptor membrane component 2	23/23
PLIN	perilipin	23/19
PTGER3	prostaglandin E receptor 3 (subtype EP3)	23/22
RPL31	ribosomal protein L31	23/23
SEMA3G	sema domain, short basic domain (semaphorin) 3G	23/20
TMEM135	transmembrane protein 135	23/23
ACAA1	acetyl-Coenzyme A acyltransferase 1	22/22
ADIPOQ	adiponectin, C1Q and collagen domain containing	22/22
ALDH1L1	aldehyde dehydrogenase 1 family, member L1	22/22
AQP7	aquaporin 7	22/19
BBOX1	(gamma-butyrobetaine hydroxylase) 1	22/22
CAMK1	calcium/calmodulin-dependent protein kinase I	22/22
CSAD	cysteine sulfinic acid decarboxylase	22/22
DHDDS	dehydrodolichyl diphosphate synthase	22/22
DTX4	deltex homolog 4 (Drosophila)	22/22
ECHDC3	enoyl Coenzyme A hydratase domain containing 3	22/22
EHBP1	EH domain binding protein 1	22/21
EPB41L4B	erythrocyte membrane protein band 4.1 like 4B	22/22
FABP4	fatty acid binding protein 4, adipocyte	22/22
GCSH	glycine cleavage system protein H (aminomethyl carrier)	22/22
GYG2	glycogenin 2	22/19
HOXA9	homeobox A9	22/22
HPGD	hydroxyprostaglandin dehydrogenase 15-(NAD)	22/22
ISOC1	isochorismatase domain containing 1	22/22

LEPR	leptin receptor	22/22
LEPREL1	LEPREL1 collagen prolyl hydroxylase	22/22
LOC730107	similar to Glycine cleavage H protein, mitochondrial	22/22
LPL	lipoprotein lipase	22/22
PEG3	paternally expressed 3	22/22
PPFIBP2	PTPRF interacting protein, (liprin beta 2)	22/22
RPL23AP13	ribosomal protein L23a pseudogene 13	22/22
RREB1	ras responsive element binding protein 1	22/22
SPINK5	serine peptidase inhibitor, Kazal type 5	22/22
UNG	uracil-DNA glycosylase	22/22

Table S2: The microarray datasets that were analyzed to generate a meta-signature

Study	Authors	PMID	Array type	Number of samples
GSE6481	Nakayama et al.	17464315	Hgu133a	105
E-MEXP-353	Hendersen et al.	16168083	Hgu133a	96

Table S3: Differential expression classes

Study	Class 1 (# of samples)	Class 2 (# of samples)
GSE6481	MLS (7)	Osteosarcoma (11)<br>Alveolar Rhabdomyosarcoma (4)<br>Chondroblastoma (4)<br>Chondromyxoid Fibroma (4)<br>Chondrosarcoma (7)<br>Chordoma (4)<br>Dedifferentiated Chondrosarcoma (3)<br>Embryonal Rhabdomyosarcoma (3)<br>Fibromatosis (5)<br>Leiomyosarcoma (8)<br>Lipoma (3)<br>Malignant Peripheral Nerve Sheath Tumor (4)<br>Monophasic Synovial Sarcoma (10)<br>Neurofibroma (4)<br>Sarcoma (3)<br>Schwannoma (4)<br>Well-differentiated Liposarcoma (3)
E-MEXP-353	MLS(19)	Dedifferentiated liposarcoma (15)<br>Fibrosarcoma (4)<br>Leiomyosarcoma (6)<br>Lipoma (3)<br>Malignant.fibrous.histiocytoma (21)<br>Malignant.peripheral.nerve.sheath.tumor (3)<br>Myxofibrosarcoma (15)<br>Synovial.sarcoma (16)<br>Well-differentiated.liposarcoma (3)

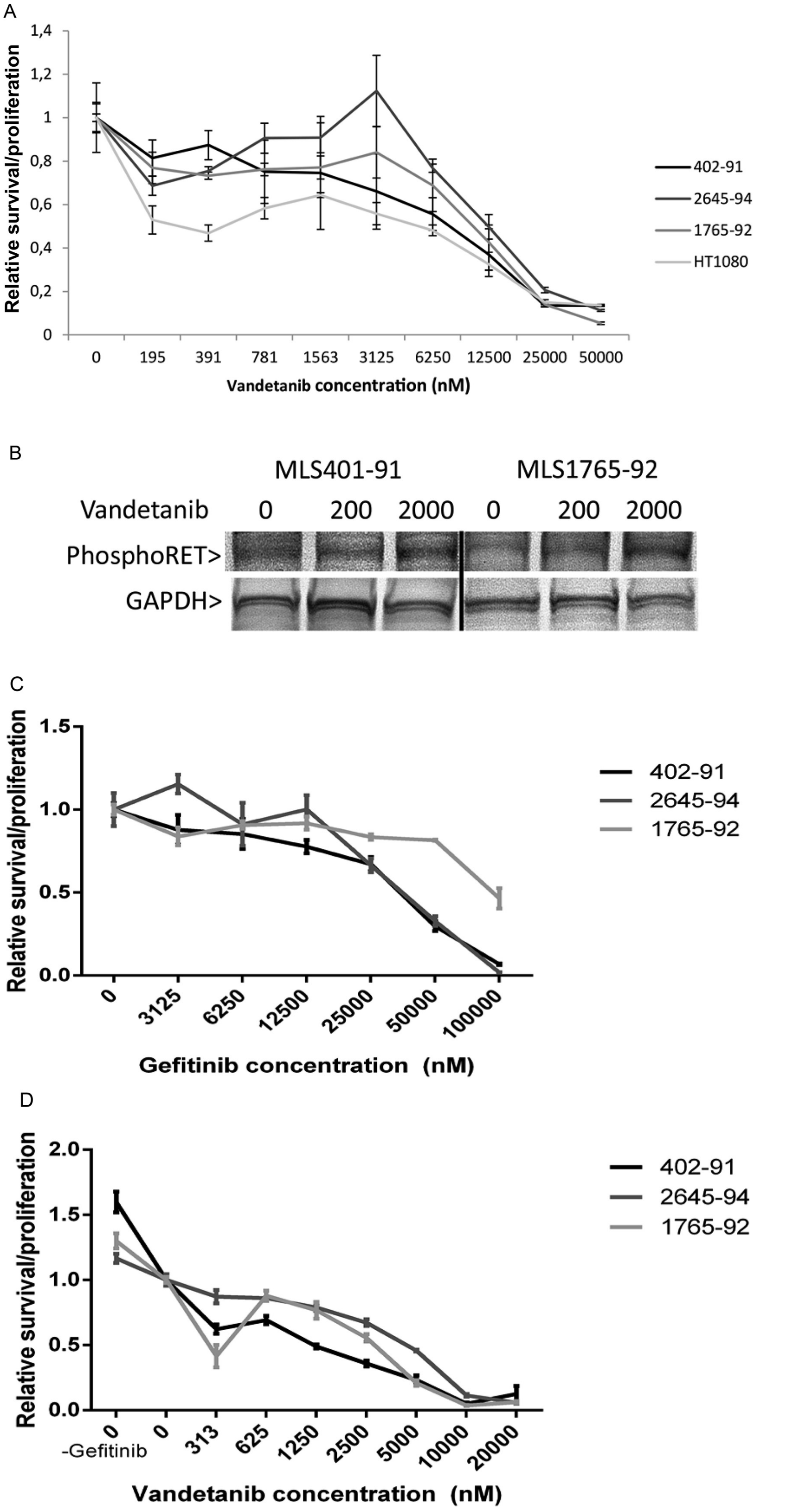

Supplementary Figure S1: Growth/suvival MLS cell lines titration Vandetanib. (**A**) Growth/survival assay of indicated cell lines with up to 50,000 nM of Vandetanib. (**B**) RET phosphorylation in MLS cell lines treated with Vandetanib. Western blot analysis of RET Y905 phosphorylation performed in whole cell lysates as described in Olofsson et al 2004. Vandetanib concentrations in nM. (**C**) Growth/survival assay of indicated cell lines with up to 1,00,000 nM Gefitinib. (**D**) Growth/survival assay with up to 20,000 nM of Vandetinib in presence of 25,000 nM of Gefitinib.

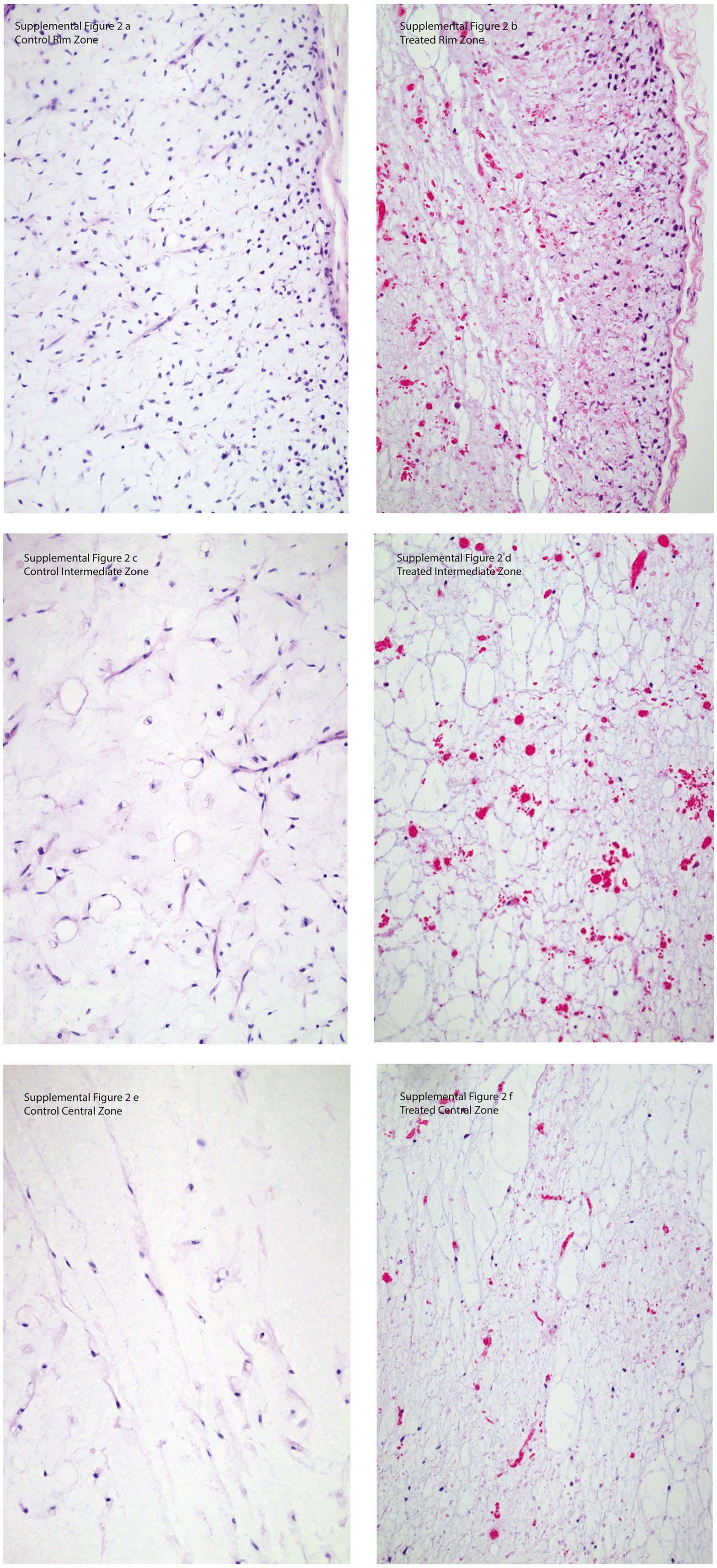

Supplementary Figure S2: Eosin-hematoxylin stained sections of xenografted MLS tumor tissues. Samples from control mouse and 17-DMAG treated animals as indicated. Pictures captured with a 20× objective and a ProGres® digital microscope camera.

Supplementary Film Clips: 3D photographs of PLA signals (red dots) from single ERBB3, RET and combined ERBB3 and RET antibodies in MLS 402.91 cells. Original photos were captured using a Zeiss 700 confocal system. DNA (nuclei) were stained with DAPI (Blue). Film clips were created with the Volocity software. The DAPI signal was faded in order to show the intranuclear PLA signals.

REFERENCES

1. Smyth GK. limma: Linear Models for Microarray Data. In: Gentleman R, Carey VJ, Huber W, Irizarry RA, Dudoit S, editors. Bioinformatics and Computational Biology Solutions Using R and Bioconductor. Springer New York. 2005; 397–420.
2. Storey JD. A direct approach to false discovery rates. J R Stat Soc Ser B Stat Methodol. 2002; 64:479–98.
3. Rhodes DR, Yu J, Shanker K, Deshpande N, Varambally R, Ghosh D, et al. Large-scale meta-analysis of cancer microarray data identifies common transcriptional profiles of neoplastic transformation and progression. Proc Natl Acad Sci USA. 2004; 101:9309–14.
